# Rescue multihole metal stent placement to prevent stent migration after endoscopic ultrasound-guided hepaticogastrostomy

**DOI:** 10.1055/a-2792-9907

**Published:** 2026-02-17

**Authors:** Akane Hara, Kosuke Minaga, Yasuo Otsuka, Mamoru Takenaka, Masatoshi Kudo

**Affiliations:** 1 Department of Gastroenterology and Hepatology, Kindai University Faculty of Medicine, Sakai, Japan

**Fig. 1:**
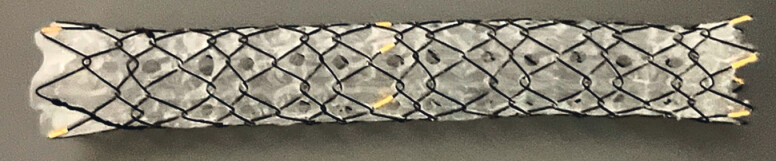
The MHSEMS (HANAROSTENT Biliary Multi-hole NEO) is a fully covered metal stent with multiple small side holes, 1.8 mm in diameter, placed on the silicone covering membrane. MHSEMS, multihole self-expandable metal stent.

During endoscopic ultrasound-guided hepaticogastrostomy (EUS-HGS), covered self-expandable metal stents (SEMSs) are preferred for durable biliary drainage; however, stent migration remains an unavoidable complication 1 2 . Multihole SEMSs (MHSEMSs), which are fully covered metal stents equipped with multiple, small side holes on the covering membrane, are designed to allow tissue ingrowth, thereby enhancing the anchoring effect to prevent stent migration and minimizing the risk of branch bile duct occlusion ( 3 , Fig. 1 ). Although MHSEMS placement during endoscopic retrograde cholangiopancreatography for distal malignant biliary obstruction (MBO) is associated with a low risk of stent migration 4 5 , its use during EUS-HGS remains unexplored.

**Fig. 2:**
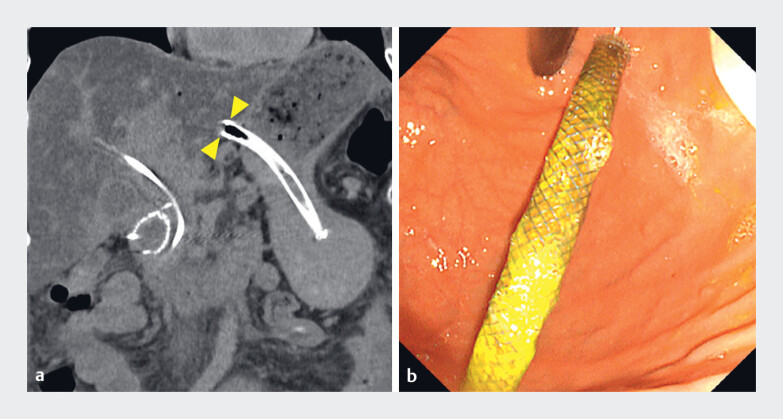
Migration of the covered metal stent toward the gastric lumen after its placement during endoscopic ultrasound-guided hepaticogastrostomy. **a** Computed tomography showing the migration of the stent into the gastric lumen. **b** An upper endoscopic view confirming the gastric migration of the stent.

**Fig. 3:**
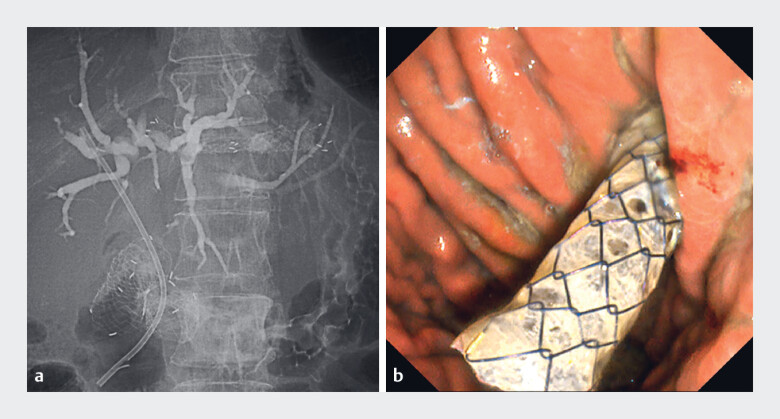
Rescue reintervention using a MHSEMS. **a** A fluoroscopic view showing the deployment of the MHSEMS bridging the intrahepatic bile duct and stomach through the fistula. **b** An endoscopic view confirming the successful placement of the MHSEMS. MHSEMS, multihole self-expandable metal stent.

**Fig. 4:**
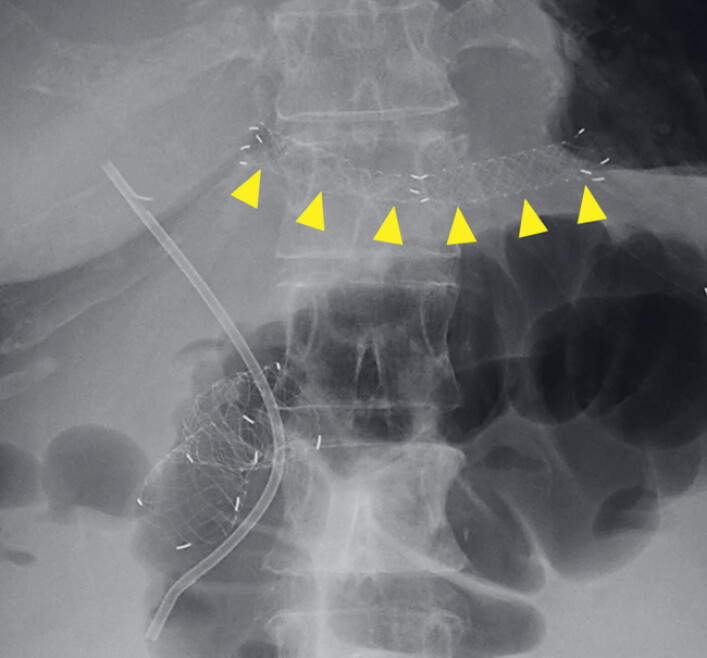
An abdominal X-ray 5 months after the MHSEMS placement showing no stent migration. MHSEMS, multihole self-expandable metal stent.

A 67-year-old woman with advanced pancreatic cancer, in whom a plastic stent was previously placed for distal MBO, subsequently developed recurrent cholangitis. As transpapillary re-intervention was not feasible because of an enteral stent traversing the duodenal papilla, EUS-HGS was performed using a partially covered SEMS. She developed recurrent cholangitis 2 months later, and computed tomography and upper endoscopy revealed the migration of the EUS-HGS stent toward the gastric lumen ( Fig. 2 ). A 7-Fr plastic stent was urgently placed through the EUS-HGS fistula; however, cholangitis recurred 1 month later due to stent dysfunction. Given the early migration of the partially covered SEMS and early plastic stent occlusion, rescue reintervention using the MHSEMS was planned. A guidewire was advanced through the fistula into the bile duct alongside the plastic stent, and the plastic stent was removed. After cholangiography, an 8-mm × 80-mm MHSEMS (HANAROSTENT Biliary Multi-hole NEO; M.I. Tech, Pyeongtaek, South Korea) was successfully deployed ( Fig. 3 ; Video 1 ). Cholangitis resolved within 4 days, and cholangitis or stent migration did not recur for 5 months until death from disease progression ( Fig. 4 ). This case illustrates the utility and safety of MHSEMSs as a rescue option for reintervention after stent migration following EUS-HGS.

The rescue placement of a multihole self-expandable metal stent to prevent stent migration after endoscopic ultrasound-guided hepaticogastrostomy.Video 1

Endoscopy_UCTN_Code_CCL_1AZ_2AB Endoscopy_UCTN_Code_TTT_1AS_2AH
